# Percentage fractions of urinary di(2-ethylhexyl) phthalate metabolites: Association with obesity and insulin resistance in Korean girls

**DOI:** 10.1371/journal.pone.0208081

**Published:** 2018-11-27

**Authors:** Shin-Hye Kim, Ji-won On, Heesoo Pyo, Kyung Soo Ko, Jong Chul Won, Jiyeon Yang, Mi Jung Park

**Affiliations:** 1 Department of Pediatrics, Inje University Sanggye Paik Hospital, Inje University College of Medicine, Seoul, Republic of Korea; 2 Molecular Recognition Research Center, Korea Institute of Science and Technology, Seoul, Republic of Korea; 3 Department of Chemistry, Korea University, Seoul, Republic of Korea; 4 Department of Internal Medicine, Inje University Sanggye Paik Hospital, Inje University College of Medicine, Seoul, Republic of Korea; 5 Institute for Environmental Research, Yonsei University College of Medicine, Seoul, Republic of Korea; Universidad de Malaga, SPAIN

## Abstract

**Objective:**

We assessed the associations of percentage fractions of urinary di(2-ethylhexyl) phthalate (DEHP) metabolites with obesity and insulin resistance in Korean girls.

**Methods:**

In total, 137 girls, aged 6 to 13 years (65 overweight cases and 72 controls), were recruited. Anthropometric indices and the homeostatic model assessment of insulin resistance (HOMA-IR) index were determined. Four major urinary DEHP metabolites were analyzed in spot urine samples by gas chromatography-tandem mass spectrometry, including mono(2-ethylhexyl) phthalate, mono(2-ethyl-5-hydroxyhexyl) phthalate (MEHHP), mono(2-ethyl-5-oxohexyl) phthalate (MEOHP), and mono(2-ethyl-5-carboxypentyl) phthalate.

**Results:**

There were no significant differences in the urinary concentrations of the DEHP metabolites between the overweight and control groups. The percentage fraction of MEHHP (MEHHP%) among all DEHP metabolites was significantly higher in the overweight prepubertal girls than in the controls (*P* = 0.035). MEHHP% was positively associated with the body mass index percentile, waist circumference, body fat percentage, and HOMA-IR index in the prepubertal girls. After adjusting for covariates, the prepubertal girls in a higher MEHHP% quartile were found to have a higher odds ratio for central obesity than those in a lower quartile (odds ratios: 5.05 for quartile 3; 7.30 for quartile 4). The relative rate of MEHHP oxidation to MEOHP was negatively associated with the body mass index percentile and waist circumference in the prepubertal girls. However, no such association was observed in the pubertal girls.

**Conclusions:**

MEHHP% was positively associated with obesity and insulin resistance in prepubertal girls. Further studies are necessary to elucidate the causal links between altered phthalate metabolism and increased susceptibility to insulin resistance in children.

## Introduction

**Fig 1:**
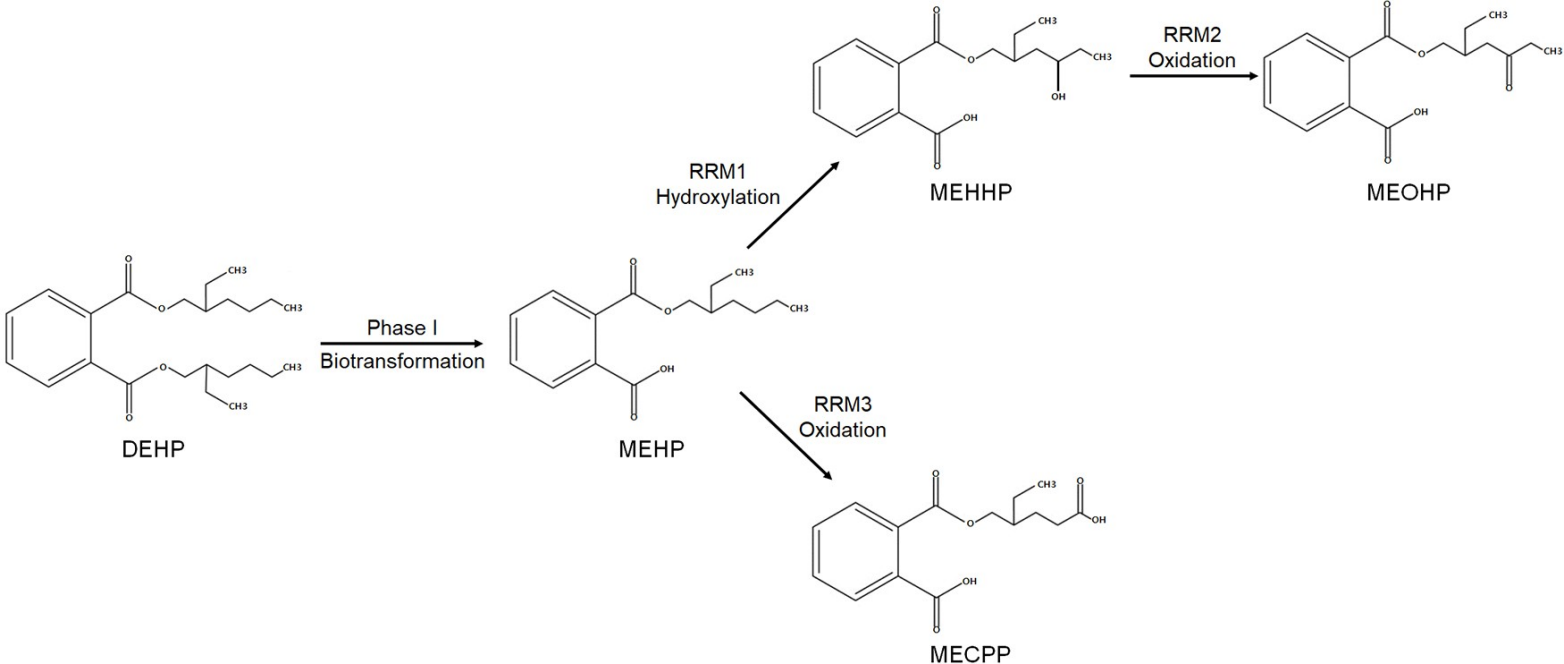
Metabolic pathway of di(2-ethylhexyl) phthalate (DEHP) metabolites. The relative rate of metabolism (RRM) was calculated as the product-to-precursor molar concentration ratio as follows: RRM1 = ([MEHHP] + [MEOHP])/[MEHP]; RRM2 = ([MEOHP]/[MEHHP]) × 10; RRM3 = [MECPP]/[MEHP].

Phthalates are 1,2-benzenedicarboxylic acid esters used as plasticizers to make plastics more flexible and soft and as vehicles in cosmetics [1]. They are found in various consumer products, including food packaging, toys, medical devices, building materials, and cosmetics [1]. Human exposure to phthalates occurs primarily through ingestion of contaminated food and water, as well as through dermal contact and air inhalation. One of the types of phthalates most frequently used in consumer products, di(2-ethylhexyl) phthalate (DEHP), is found at relatively high concentrations in human specimens including blood, urine, breast milk, and feces, and its metabolism has been widely studied [2]. The metabolic pathway of DEHP involves a series of chemical reactions that produce various metabolites [3]. Following exposure, DEHP is rapidly hydrolyzed by esterases in the gut, liver, and blood into mono(2-ethylhexyl) phthalate (MEHP), which is subsequently metabolized by hepatic and intestinal cytochrome P450 (CYP) enzymes into various secondary oxidative metabolites. Those secondary oxidative metabolites include mono(2-ethyl-5-hydroxyhexyl) phthalate (MEHHP), mono(2-ethyl-5-oxohexyl) phthalate (MEOHP), and mono(2-ethyl-5-carboxypentyl) phthalate (MECPP) (Fig 1). In human oral exposure study, approximately 75% of the DEHP dose was excreted in urine in the form of the DEHP metabolites after 44 hours [3]. Therefore, the measured urinary concentrations of the oxidative DEHP metabolites are commonly used as biomarkers of exposure to DEHP [1, 3].

It has been suggested that phthalates are obesogens and may induce obesity and metabolic derangement [1]. This hypothesis has been supported by previous animal studies showing that phthalate exposure increases body weight and insulin resistance [4–6]. Insulin resistance is defined as an impairment in the ability of peripheral tissues to take up and utilize glucose in response to insulin action, which leads to hyperinsulinemia. Insulin resistance is strongly associated with central (visceral) obesity and an increased risk of metabolic syndrome and type 2 diabetes. The possible mechanisms of the development of obesity/insulin resistance upon phthalate exposure have been suggested as follows: activation of peroxisome proliferator-activated receptors, inhibition of thyroid hormone and androgens, and direct interference with the expression of the insulin receptor gene [7].

In recent decades, the relationship between DEHP exposure and obesity/insulin resistance has been investigated in human studies [8–13]. However, the results of these studies have been inconsistent and depended on the subjects’ demographic characteristics. Two U.S. epidemiological studies based on the National Health and Nutrition Examination Survey data have demonstrated positive associations of urinary DEHP metabolite concentrations with waist circumference (WC) and obesity in U.S. adults [8, 12], whereas another study reported a negative association between the concentration of a specific urinary DEHP metabolites and the body mass index (BMI) in U.S. elderly women [9]. Regarding the insulin resistance, one previous study did not find any significant relationship of urinary DEHP metabolite concentrations with the homeostasis model assessment of insulin resistance (HOMA-IR) index in U.S. adult men [12], whereas more recent studies have found positive associations in U.S. adults [10], Belgian adults with obesity [13], and a Korean elderly population [11]. It has been suggested that the vulnerability to phthalates in regard to insulin resistance may be different, depending on ethnicity and gender; however, the possible mechanism has not yet been adequately explained [9].

Previous studies have shown that percentage fractions of secondary metabolites of DEHP, which may reflect the patterns of DEHP metabolism in organisms, differ, depending on the species and age of laboratory animals [14, 15]. Similarly, in humans, the ratios of DEHP metabolites have been reported to differ, depending on the subjects’ demographic factors. For instance, higher ratios between MEHHP/MEOHP in European population were noted compared with those in people from USA, Taiwan, and Israel [16]. The rate of MEHP-to-MEHHP metabolic conversion in infants and children was reported to be faster than that in adults [17, 18]. Therefore, we speculated that the inconsistent results of previous studies on the relationship between urinary DEHP metabolites and obesity/insulin resistance might be partly explained by differences in the rate of DEHP metabolism.

In this study, we assessed whether the concentrations of individual DEHP metabolites and their percentage fractions are related to obesity indices and insulin resistance in girls.

## Subjects and methods

### Subjects and study design

In total, of 65 overweight girls and 72 age-matched controls, aged 6–13 years, were recruited from the Inje University Sanggye Paik Hospital (Seoul, Korea) between March 2015 and September 2015. The overweight girls were recruited from patients who visited the obesity clinic to seek medical assessment. As controls, non-overweight girls were enrolled from those who visited the pediatric health clinic for regular check-ups related to growth and development. All eligible girls who attend the obesity clinic and pediatric health clinic during the study period were asked whether they would like to participate in the study. The eligibility of the subjects was identified before recruitment. All of the subjects recruited in this study were from Seoul, the metropolitan city in South Korea. All participants voluntarily visited the hospital without being referred from the local primary health care providers, devoid of a history of exposure to Polyvinyl Chloride (PVC)- containing medical devices through intravenous procedures during the previous one month. They were assessed via physical examination and history taking to exclude underlying chronic diseases, endocrinopathies, and possible use of medications. Individuals who had been diagnosed with medical problems, including diabetes, thyroid disease, hepatitis, familial hypercholesterolemia, or epilepsy requiring anticonvulsive therapy, were excluded. The study protocol was approved by the Institutional Review Board of the Inje University Sanggye Paik Hospital (SGPAIK 2015-01-001-001), and written informed consent was obtained from all subjects under age 18 and their parents before enrollment.

### Anthropometric measurements and pubertal staging

The body weight and body fat percentage were measured using an InBody 720 body composition analyzer (Biospace Co., Ltd., Seoul, Korea), and the height was measured using a stadiometer (Dongsahn Jenix Co., Seoul, Korea). The BMI (kg/m^2^) of each subject was calculated based on these measures. The WC measurement was taken at the midpoint between the highest point of the iliac crest and the lowest point of the rib cage, at the end of a normal expiration, with subjects standing in an upright position. Age- and gender-specific percentiles of the height, weight, and BMI were calculated for each subject based on national reference charts [19]. Overweight was defined as BMI ≥ 85^th^ percentile, and central obesity was defined as WC ≥ 90^th^ percentile values for the age and gender.

Because insulin resistance increases in the pubertal period, sexual maturation of the breast was determined in all subjects by a pediatric endocrinologist according to the Tanner staging system [20]. The Tanner scale defines five sequential stages of sexual maturation, from prepuberty (stage 1) to adulthood (stage 5). Stage 2 indicates the onset of secondary sexual development and the beginning of puberty; therefore, subjects who had reached at least stage 2 of breast development were grouped as pubertal girls, whereas those who had not reached this stage were classified as prepubertal girls.

### Analysis of urinary DEHP metabolites

First morning spot urine samples were collected in polyethylene cups following a 10-h overnight fast and stored at −80 °C until analysis. The sample preparation methods were described elsewhere [18]. Four DEHP metabolites (MEHP, MEHHP, MEOHP, and MECPP) were analyzed by gas chromatography-tandem mass spectrometry using a 7890A gas chromatograph and 7000 triple quadrupole mass spectrometer (Agilent, Palo Alto, CA, USA), with preceding enzymatic deconjugation, followed by liquid-liquid extraction. In brief, isotope-labeled internal standards mixture was spiked into the urine sample. The spiked sample was then buffered with ammonium acetate (1 M, pH = 6.0) and enzymatically hydrolyzed with *E-coli* β-glucuronidase at 37 °C for 2 h. Deconjugated sample was then buffered with 2 M acetic acid at pH 4.0–4.5 followed by liquid-liquid extraction with hexane-ether solvents (8:2, v/v). The mixture was centrifuged, and the organic phase was separated with freezer, concentrated under nitrogen and dehydrated in vacuum desiccators. Trimethylsilyl derivatization was employed with BSFTA/TMCS (99: 1, v/v) at 65 °C for 30 min and resulting derivatives were analyzed by gas chromatography-tandem mass spectrometry using isotope dilution. We evaluated recovery and precision by spiking standard material in reagent water. When the spiked concentration in the sample (n = 5) was 10 ppb, recovery and precision were in the range of 61.61%–100.11% and 4.27%–6.53%, respectively. The pooled urine samples (n = 5) were analyzed for each batch to perform the internal quality assurance/quality control (QA/QC), and the precision ranged from 4.76% to 16.32% for the DEHP metabolites studied. The limits of detection for MEHP, MEHHP, MEOHP, and MECPP were 0.2, 0.5, 1.0, and 5.0 μg/L, respectively. Detailed analytical method was provided in S1 Fig.

We presented the urine concentrations of DEHP metabolites in three forms, before correction (μg/L), urine creatinine-corrected concentrations (μg/g Cr), and urine specific gravity (SG)-corrected concentrations (μg/L). A creatinine-corrected concentration is a value of the measured urinary DEHP concentration (μg/L) divided by the urine creatinine level (g/L), which is the most common method to adjust the urinary dilution in biomonitoring urinary chemical concentrations. However, several physiological parameters including age, renal function, and muscle mass influence urine creatinine concentrations. Therefore, we also used the SG-corrected concentrations, which is not influenced by age and muscle mass. The correction formula is Pc = P x [(1.024–1)/(SG-1)], where Pc is the SG-corrected DEHP metabolite concentrations (μg/L), and P is the experimental DEHP metabolite concentrations [21].

Percentage fractions of the DEHP metabolites were calculated as a percentage ratio of the molar concentration of a specific metabolite to the sum of the molar concentrations of all DEHP metabolites. For example, MEHP% was determined as the ratio of [MEHP]/([MEHP] + [MEHHP] + [MEOHP] + [MECPP]) × 100. We also calculated the relative rate of metabolism (RRM) to estimate the production rate of DEHP metabolites from their precursors. RRMs were calculated as the product-to-precursor molar concentration ratios as follows: RRM1, ([MEHHP] + [MEOHP])/[MEHP]; RRM2, ([MEOHP]/[MEHHP]) × 10; and RRM3, [MECPP]/[MEHP] (Fig 1). Thus, RRM1 represents the rate of hydroxylation of MEHP to MEHHP, whereas RRM2 and RRM3 represent the rates of oxidation from MEHHP to MEOHP and from MEHP to MECPP, respectively.

### Laboratory tests for insulin resistance

Blood samples were obtained on the day of urine sampling, following a 10-h overnight fast. Plasma glucose levels were measured using an enzymatic assay (Pureauto S GLU; Daiichi, Tokyo, Japan), and serum insulin levels were measured using an immunoradiometric assay (INS-Irma; Biosource, Nivelles, Belgium). Insulin resistance in the subjects was estimated using the HOMA-IR index, which is the most commonly used surrogate marker for insulin resistance in children. HOMA-IR shows a good correlation with the hyperinsulinemic–euglycemic clamp test, which is the gold standard for the measurement of insulin resistance. The HOMA-IR index was calculated as fasting glucose (mg/dL) × fasting insulin (μU/mL)/405 [22].

### Statistical analysis

Based on the study results of Wang et al. [21] on the DEHP metabolites concentrations of between overweight and control groups, the sample size was calculated by setting the mean differences of urinary MEHP levels (2.8 ng/mL), standard deviation (1.1), 5% level of significance, and 95% statistical power. The concentrations of the phthalate metabolites and insulin, as well as the HOMA-IR index, were log-transformed to improve the approximation of the normal distribution. Data are shown as mean ± standard errors or number (%), unless otherwise indicated. Data were analyzed using independent *t*-tests and chi-squared tests for continuous variables and discrete variables, respectively. Multiple linear regression was used to explore the relationships between phthalate metabolite concentrations and obesity-related metabolic parameters, after adjustment for age, Tanner stage, and height/BMI percentile. Estimated differences in the means and 95% confidence interval (CI) were calculated using analysis of covariance to compare anthropometric data and metabolic parameters, including the HOMA-IR index, with the MEHHP% quartile. The *P*-for-trend was obtained by linear regression modeling of the MEHHP% quartile as a continuous variable. Multiple logistic regression analysis was performed to examine the odds ratio (OR) and 95% CI for central obesity across the MEHHP% quartile, after adjusting for the age and height percentiles. Statistical analyses were performed using PASW Statistics for Windows, version 18.0 (SPSS, Inc., Chicago, IL, USA). Two-tailed *P* < 0.05 was considered statistically significant.

## Results

The general characteristics of the subjects are summarized in Table 1. The mean ages of the prepubertal and pubertal girls were 8.1 and 9.5 years, respectively. The overweight girls had a significantly higher BMI, WC, body fat percentage, and HOMA-IR index than the prepubertal and pubertal control girls.

**Table 1:** General characteristics of the study subjects by obesity and the puberty status.

	Prepubertal girls			Pubertal girls			*P-*value (pubertal vs prepubertal girls)
	Control	Overweight	*P*-value	Control	Overweight	*P*-value	
	n = 35	n = 33		n = 37	n = 32		
Age (years)	8.2 ± 0.1	8.0 ± 0.1	0.596	9.7 ± 0.2	9.3 ± 0.3	0.277	< 0.001
BMI (kg/m^2^)	16.5 ± 0.2	21.7 ± 0.3	< 0.001	16.8 ± 0.2	22.2 ± 0.4	< 0.001	0.580
BMI percentile	49.4 ± 4.0	95.8 ± 0.7	< 0.001	42.3 ± 3.3	94.8 ± 0.7	< 0.001	0.296
WC (cm)	59.5 ± 1.0	72.2 ± 1.1	< 0.001	61.9 ± 1.0	74.6 ± 1.5	< 0.001	0.353
WC percentile	61.2 ± 4.8	95.5 ± 0.8	< 0.001	53.9 ± 4.2	93.0 ± 1.9	< 0.001	0.158
Body fat (%)	21.1 ± 1.3	35.4 ± 0.8	< 0.001	22.9 ± 0.9	32.7 ± 1.5	< 0.001	0.544
Tanner stage						0.364	< 0.001
1	35 (100%)	33 (100%)		0 (0%)	0 (0%)		
2				21 (56.8%)	13 (40.6%)		
3				7 (18.9%)	12 (37.5%)		
4				7 (18.9%)	5 (15.6%)		
5				2 (5.4%)	2 (6.3%)		
Fasting glucose (mg/dL)	92.4 ± 1.0	96.8 ± 1.1	0.007	92.7 ± 1.3	94.3 ± 1.6	0.468	0.379
Fasting insulin (μU/mL)	8.1 ± 0.7	12.9 ± 1.1	0.001	9.8 ± 0.8	17.0 ± 2.7	0.006	0.041
HOMA-IR	1.9 ± 0.1	3.1 ± 0.2	0.001	2.3 ± 0.1	3.8 ± 0.5	0.007	0.105

The distributions of the urinary DEHP metabolites are presented in Table 2 and S2 Fig. All metabolite concentrations were above the limits of detection. Among the DEHP urinary metabolites, MECPP was detected at the highest concentration, followed by MEHHP, MEOHP, and MEHP. The percentage fraction was also the highest for MECPP% (50.3%) among the individual DEHP metabolites, followed by MEHHP% (22.5%), MEOHP% (17.9%), and MEHP% (9.4%).

**Table 2:** Geometric means (GM) and selective percentiles of urinary phthalate metabolite concentrations.

Phthalate metabolites	Range	GM	Percentile				
			10th	25th	50th	75th	90th
**Uncorrected concentrations (μg/L)**							
MEHP	0.3–54.0	11.1	6.3	7.7	10.2	15.7	23.2
MEHHP	4.2–378.9	31.8	9.9	16.1	33.6	57.5	95.0
MEOHP	4.7–194.8	25.5	9.1	13.5	24.9	45.7	69.4
MECPP	5.0–919.6	75.3	19.8	41.4	78.6	146	230
**Specific gravity-corrected concentrations (μg/L)**							
MEHP	0.2–85.5	15.4	7.9	9.9	14.9	23.1	39.5
MEHHP	6.3–413.4	44.2	18.0	27.4	42.2	73.4	123
MEOHP	6.7–287.2	35.4	15.7	21.7	32.7	57.1	92.8
MECPP	14.3–1127.5	104	41.4	59.5	103	163	345
**Creatinine-corrected concentrations (μg/g Cr)**							
MEHP	0.2–75.6	12.6	6.7	9.7	12.6	19.6	32.1
MEHHP	7.1–533	36.8	17.8	26.8	36.5	56.7	83.2
MEOHP	7.3–274	29.5	14.5	21.1	30.6	45.1	65.8
MECPP	18.8–940	87.3	37.3	55.5	90.8	140	206

The concentrations and percentage fractions of the urinary DEHP metabolites are stratified by the pubertal and obesity statuses in Table 3. There were no significant differences in the urinary DEHP metabolite levels between the overweight and control girls, regardless of the pubertal status. Among the prepubertal girls, a significantly higher MEHHP% was found in the overweight girls than in the controls (Table 3 and S2 Fig), whereas MEHP%, MEOHP%, and MECPP% were not significantly different depending on the obesity status. In the pubertal girls, no significant differences were identified in the percentage fractions of the urinary DEHP metabolites between the overweight and control groups.

**Table 3:** Geometric means (± standard error) of urinary phthalate metabolite concentrations and percentage fractions of di(2-ethylhexyl) phthalate (DEHP) metabolites by obesity and puberty status.

	Prepubertal girls			Pubertal girls			*P-*value (pubertal vs prepubertal girls
	Control	Overweight	*P*-value	Control	Overweight	*P*-value	
	n = 35	n = 33		n = 37	n = 32		
**Uncorrected concentrations (μg/L)**							
MEHP	13.9 ± 1.7	12.9 ± 1.6	0.679	14.1 ± 1.4	10.9 ± 0.7	0.058	0.562
MEHHP	44.7 ± 6.4	49.3 ± 11.7	0.732	47.8 ± 6.2	44.8 ± 10.3	0.801	0.947
MEOHP	36.6 ± 5.1	34.6 ± 6.5	0.805	37.5 ± 5.0	31.6 ± 5.6	0.435	0.876
MECPP	121.6 ±	119.7 ± 26.1	0.950	121.4 ± 20.3	111.0 ± 27.9	0.761	0.867
**Specific gravity-corrected concentrations (μg/L)**							
MEHP	20.3 ± 2.7	19.6 ± 3.0	0.854	20.4 ± 2.1	15.8 ± 1.5	0.089	0.489
MEHHP	55.2 ± 7.2	66.0 ± 15.9	0.529	63.0 ± 7.1	54.7 ± 10.2	0.494	0.905
MEOHP	45.8 ± 5.6	48.1 ± 58.1	0.843	50.1 ± 5.8	40.6 ± 5.9	0.261	0.861
MECPP	144.4 ± 21.6	159.6 ± 40.5	0.737	161.5 ± 23.1	136.1 ± 28.4	0.486	0.944
**Creatinine-corrected concentrations (μg/g Cr)**							
MEHP	15.2 ± 2.5	14.0 ± 2.9	0.848	11.9 ± 1.4	13.2 ± 1.5	0.913	0.080
MEHHP	41.5 ± 5.6	38.3 ± 15.6	0.941	37.7 ± 4.3	37.7 ± 5.8	0.994	0.350
MEOHP	35.0 ± 4.5	29.7 ± 8.1	0.640	30.3 ± 3.5	29.7 ± 3.4	0.741	0.243
MECPP	104.1 ± 1.7	82.8 ± 29.3	0.989	90.3 ± 14.4	90.9 ± 15.6	0.895	0.413
**% of DEHP metabolites (based on molarity)**							
MEHP%	9.4 ± 0.9	10.2 ± 1.1	0.560	8.6 ± 0.6	9.4 ± 0.9	0.435	0.360
MEHHP%	21.5 ± 0.5	23.5 ± 0.8	0.035	22.7 ± 0.5	22.4 ± 0.5	0.687	0.839
MEOHP%	17.9 ± 0.5	18.0 ± 0.6	0.916	17.9 ± 0.3	17.5 ± 0.4	0.433	0.600
MECPP%	51.2 ± 1.6	48.3 ± 1.8	0.249	50.8 ± 0.9	50.7 ± 1.1	0.948	0.500

RRMs of the DEHP metabolites were stratified by the obesity and puberty status (S1 Table). A significantly lower RRM2 was found in the overweight girls than in the controls, but only for the prepubertal girls. No significant differences were found in RRM1 and RRM2, regardless of obesity and puberty status.

**Fig 2:**
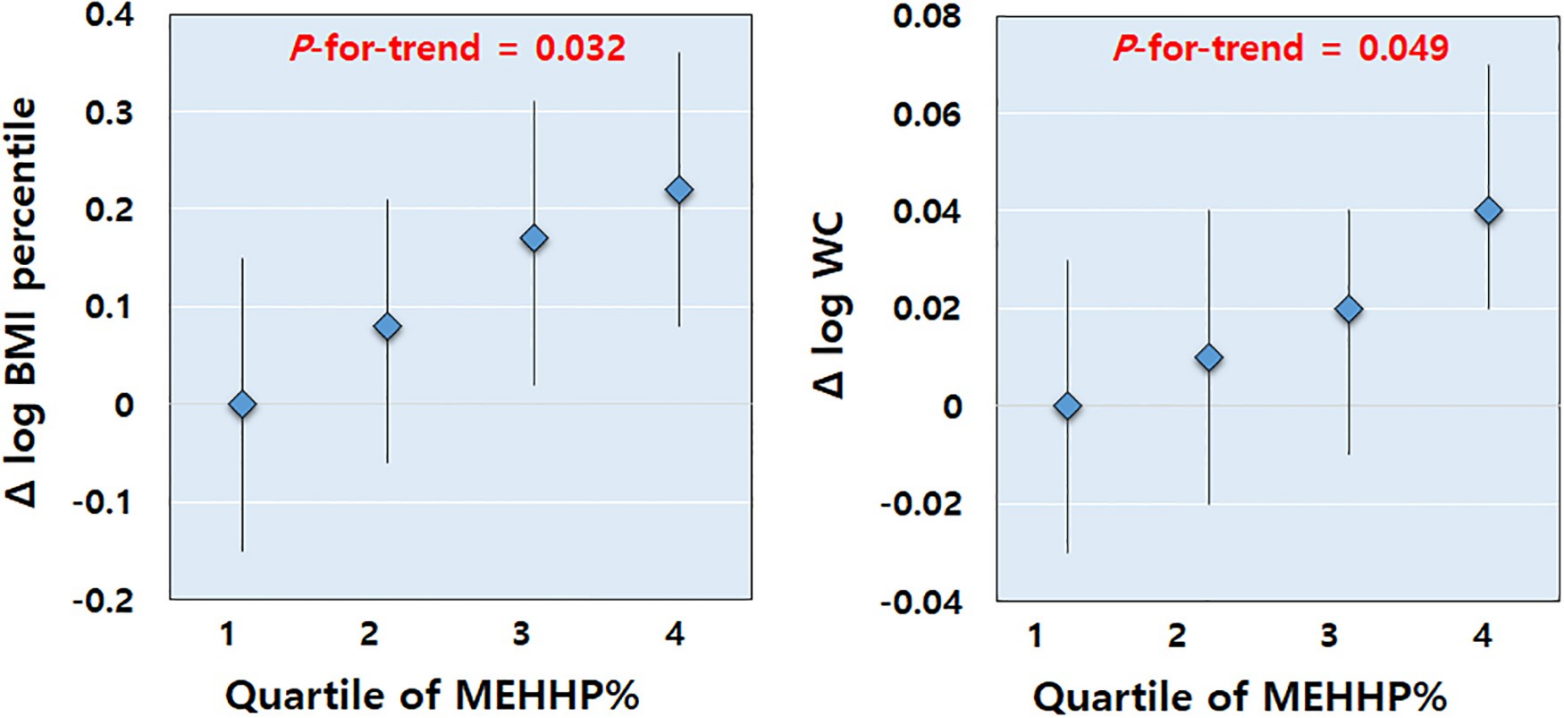
Estimated differences in the means and 95% confidence intervals of the body mass index (BMI) percentile and waist circumference (WC) stratified by the percentage fraction of mono(2-ethyl-5-hydroxyhexyl) phthalate (MEHHP%) quartiles in prepubertal girls (adjusted for age and height percentiles). For higher MEHHP% quartiles, the BMI percentile and WC increased in prepubertal girls (*P*-for-trend < 0.05).

**Fig 3:**
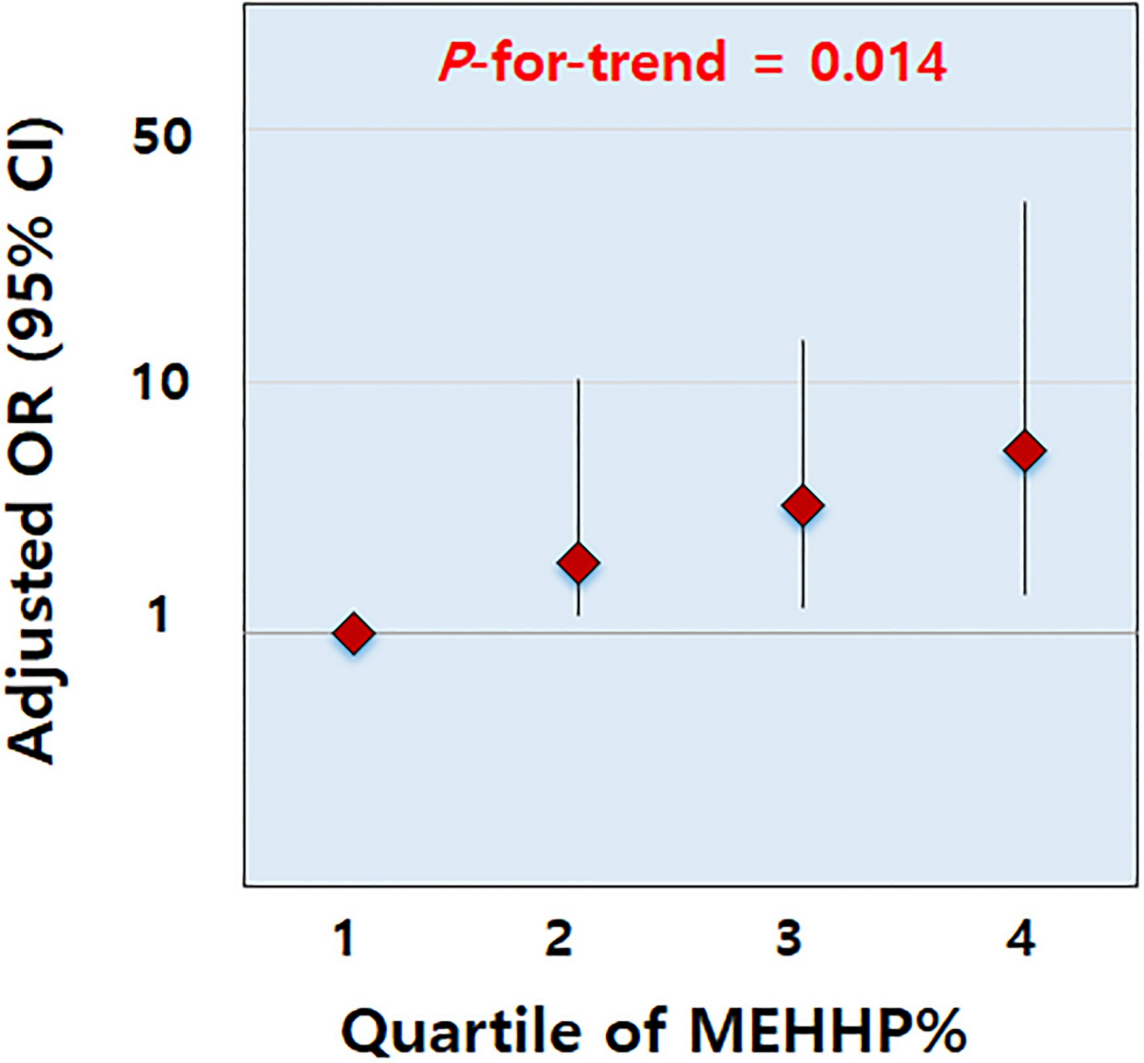
Adjusted odds ratios (ORs) and 95% confidence intervals for central obesity in prepubertal girls, stratified by percent fractions of mono(2-ethyl-5-hydroxyhexyl) phthalate (MEHHP%) quartiles (adjusted for age and height percentiles). The adjusted OR for central obesity significantly increased for higher MEHHP% quartiles (*P*-for-trend = 0.014).

After controlling for the age and height percentiles, MEHHP% was positively associated with the BMI percentile, WC, and body fat percentage in the prepubertal girls (Table 4 and Fig 2). The adjusted ORs for central obesity increased for higher MEHHP% quartiles and were 5.05 for quartile 3 and 7.30 for quartile 4 (Fig 3). In the pubertal girls, however, no significant associations were found between the percentage fractions of the DEHP metabolites and anthropometric indices. RRM2 showed a significant negative association with the BMI percentile and WC in the prepubertal girls (S2 Table). In the pubertal girls, no significant associations were found between RRMs of the DEHP metabolites and obesity indices.

**Table 4:** Regression analysis of associations between percentage fractions of di(2-ethylhexyl) phthalate (DEHP) metabolites and anthropometric indices (adjusted for age, Tanner stage, and height percentile, *P < 0.05).

	BMI percentile	WC (cm)	Body fat (%)
	β (95% CI)	β (95% CI)	β (95% CI)
**Prepubertal girls**			
MEHP%	0.65 (**−**0.56 to 1.85)	0.09 (**−**0.29 to 0.46)	**−**0.01(**−**0.42 to 0.40)
MEHHP%	1.93 (0.18 to 3.70)*	0.67 (0.15 to 1.19)*	0.60 (0.03 to 1.18)*
MEOHP%	0.23 (**−**1.98 to 2.44)	0.20 (**−**0.47 to 0.88)	0.21 (**−**0.52 to 0.94)
MECPP%	**−**0.53 (**−**1.21 to 0.16)	**−**0.52 (**−**0.09 to 0.56)	**−**1.11 (**−**0.34 to 1.12)
**Pubertal girls**			
MEHP%	0.50 (**−**0.45 to 1.47)	0.06 (**−**0.24 to 0.37)	**−**0.05 (**−**0.35 to 0.25)
MEHHP%	0.69 (**−**0.71 to 2.09)	0.37 (**−**0.06 to 0.80)	0.34 (**−**0.33 to 1.00)
MEOHP%	**−**0.36 (**−**2.17 to 1.45)	**−**0.11 (**−**0.68 to 0.45)	**−**0.08 (**−**0.48 to 0.63)
MECPP%	**−**0.28 (**−**0.88 to 0.31)	**−**0.08 (**−**0.27 to 0.10)	**−**0.08 (**−**0.27 to 0.10)

**Fig 4:**
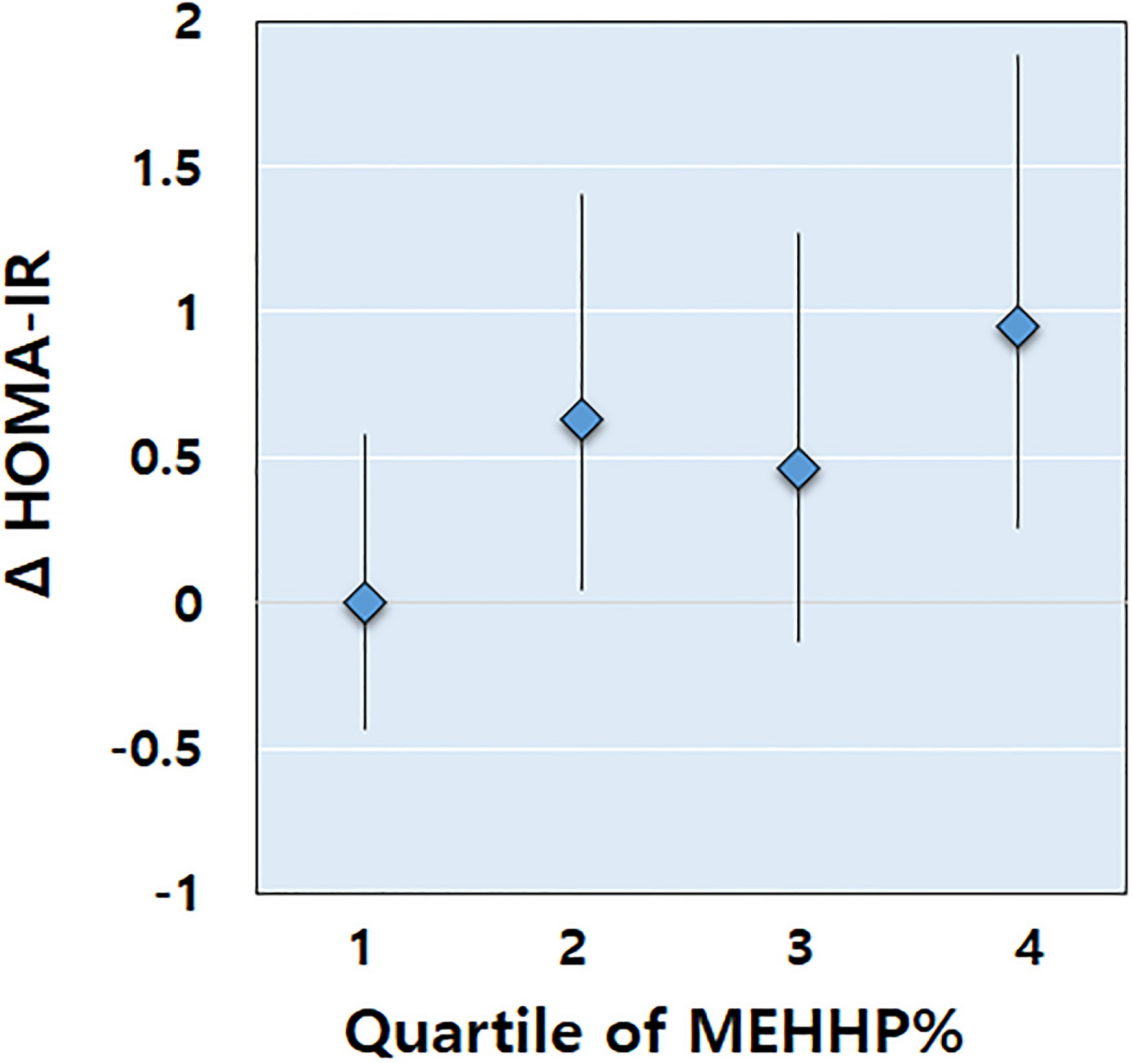
Estimated differences in the means and 95% confidence intervals of the homeostatic model assessment of insulin resistance (HOMA-IR) index stratified by quartiles of percentage fractions of mono(2-ethyl-5-hydroxyhexyl) phthalate (MEHHP%) in prepubertal girls (adjusted for age and obesity status). Prepubertal girls in the highest quartile of MEHHP% had significantly higher HOMA-IR indexes compared with those in the lowest quartile (*P* = 0.035).

The HOMA-IR indexes were positively associated with MEHHP% in the prepubertal girls, even after adjusting for the age and obesity status (Fig 4). Additionally, the urinary concentrations of the individual DEHP metabolites showed no significant associations with anthropometric indices or insulin resistance markers, regardless of puberty status (data not shown).

## Discussion

In this study, we found that obese prepubertal girls showed higher MEHHP% and lowered RRM2, suggesting an impaired rate of MEHHP-to-MEOHP metabolic conversion. MEHHP% in prepubertal girls was positively associated with obesity markers, including BMI percentile, WC, and the body fat percentage. Furthermore, MEHHP% positively correlated with insulin resistance in prepubertal girls even after adjusting for obesity status. To the best of our knowledge, this study is the first to show a positive correlation between MEHHP% and an insulin resistance marker (HOMA-IR index) in children.

In our study, the concentrations of individual DEHP metabolites and their total concentration did not show significant relationships with obesity parameters in the girls, which is consistent with the results of a large-scale cross-sectional study that used the National Health and Nutrition Examination Survey [23] and a prospective cohort study of New York City children [24]. Emerging evidence suggests that DEHP exposure, estimated by urinary DEHP concentrations, may be linked to obesity in children, but the results are inconsistent [23–25]. A positive correlation between BMI and the urinary MEHHP has been reported in prepubertal Chinese boys [21, 25], whereas an inverse correlation has been observed in prepubertal Chinese girls [25]. One study has shown a negative correlation between the urinary MECPP concentration and BMI in Danish children younger than 10 years old [26], although positive correlations have been reported between the urinary MECPP concentrations and increased BMI and WC in prepubertal and pubertal Italian children [27]. It is unclear why the associations between DEHP metabolite concentrations and childhood obesity are not consistent and depend on these demographic characteristics. One possible explanation is that these characteristics may lead to differences in the pharmacokinetics of DEHP. DEHP is metabolized in humans and laboratory animals by CYP enzymes, and previous studies have suggested that the rate of metabolism is determined by individual-specific differences in CYP enzyme activities in the liver and intestine [14, 15, 28, 29]. Furthermore, a recent *in vitro* study has indicated that human hepatic CYP2C9 is the principal isoform involved in the production of MEHP-derived metabolites, and the substrate specificity and catalytic activity of CYP2C9 differ, depending on genetic variations [28, 30, 31]. A recent study has shown that the MEHHP/MEOHP ratios of human population widely varies by race/ethnicity from the different geographical areas [16]. Therefore, genetic differences in the capacity to metabolize DEHP might partly explain the different relationships between urinary DEHP metabolites and obesity/insulin resistance in previous human studies.

Some previous studies have demonstrated positive associations between MEHP% and BMI in children and adult populations [26, 32]. Regarding the insulin resistance, a few studies have demonstrated that increased urinary DEHP metabolite concentrations were linked to insulin resistance in adults [10, 11, 13] and children [33]. However, the associations between percentage fractions of individual DEHP metabolites and anthropometric measures/insulin resistance markers have not been thoroughly investigated yet. We found that MEHHP% was positively correlated with the BMI percentile, WC, and body fat percentage in prepubertal obese girls. Furthermore, MEHHP% was related to the HOMA-IR index in prepubertal obese girls, even after adjusting for the obesity status. Of note, previous studies have revealed that endocrine-disrupting effects and toxicities of individual DEHP metabolites are not identical. MEHHP has been reported as a potential activator of peroxisomal enzymes [34] and an anti-androgen [35] in *in vitro* studies, which have not identified endocrine-disrupting effects of MECPP. Thus, our findings, along with previous studies, suggest that patterns of DEHP metabolism among individuals may be an essential determinant of health impact after DEHP exposure. We suggest that in studies on the associations between EDC exposure and health outcome, the metabolic rate of the EDCs need to be considered, mainly when the target EDCs are metabolized into multiple metabolites in the body. However, given the cross-sectional nature of this study, we could not determine a causal relationship between the decreased rate of MEHHP metabolism and the occurrence of obesity/insulin resistance.

We observed puberty-related differences in the correlations between percentage fractions of DEHP metabolites and metabolic parameters in this study. This finding may be related to the endogenous estrogen status and subsequent changes in CYP enzymatic activity [36]. Animal studies have demonstrated that female hormones, including estradiol and progesterone, may influence the rate of drug metabolism by changing hepatic CYP expression [37, 38]. Furthermore, the results of a recent experimental study suggested that estradiol may change the enzyme activity of human hepatic CYP2C9 [39], which could partly explain the differences in the association between DEHP metabolism and obesity related to puberty status or age. However, because this was a cross-sectional study that included only Korean girls, the impact of puberty on the relationship between obesity and DEHP metabolism is still an open question.

Our study had a few limitations. First, we did not assess dietary patterns and calorie intakes in the study subjects. Second, the cross-sectional design of the study limited the inference of causality. Third, we used single-spot urine samples to measure urinary DEHP metabolites. Nonetheless, this is the first attempt to correlate the percentage fractions of individual DEHP metabolites with central obesity and insulin resistance in children. Furthermore, we demonstrated a significant relationship between MEHHP% and insulin resistance/central obesity in prepubertal girls, even after controlling for the age and obesity status.

## Conclusions

In conclusion, a higher MEHHP% and lower molar concentration ratio of MEOHP/MEHHP were associated with obesity and insulin resistance in prepubertal girls. However, it remains unclear whether impaired MEHHP-to-MEOHP metabolism is a cause or consequence of childhood obesity and insulin resistance. The potential causal links between the relative rates of DEHP metabolism and metabolic perturbations in children require further investigation.

## Supporting information

S1 Table Relative metabolic rates of di(2-ethylhexyl) phthalate (DEHP) metabolites by obesity and the puberty status. (DOCX)

S2 Table Regression analysis of associations between relative metabolic rates of di(2-ethylhexyl) phthalate (DEHP) metabolites and anthropometric indices. (DOCX)

S1 Fig Gas chromatography parameters of DEHP metabolite analysis in urine samples. (TIF)

S2 Fig Percentage fractions of di(2-ethylhexyl) phthalate (DEHP) metabolites according to the subject’s obesity and pubertal status. (TIF)

## Abbreviations

BMI: body mass index
CI: confidence interval
Cr: creatinine
CYP: cytochrome P450
DEHP: di(2-ethylhexyl) phthalate
HOMA-IR: homeostatic model assessment of insulin resistance
MECPP: mono(2-ethyl-5-carboxypentyl) phthalate
MEHHP: mono(2-ethyl-5-hydroxyhexyl) phthalate
MEHP: mono(2-ethylhexyl) phthalate
MEOHP: mono(2-ethyl-5-oxohexyl) phthalate
OR: odds ratio
RRM: relative rate of metabolism
WC: waist circumference
