# Correction: Percentage fractions of urinary di(2-ethylhexyl) phthalate metabolites: Association with obesity and insulin resistance in Korean girls

**DOI:** 10.1371/journal.pone.0209724

**Published:** 2018-12-19

**Authors:** Shin-Hye Kim, Ji-won On, Heesoo Pyo, Kyung Soo Ko, Jong Chul Won, Jiyeon Yang, Mi Jung Park

The affiliation for the third author is incorrect. Heesoo Pyo is not affiliated with #3 but with #2 Molecular Recognition Research Center, Korea Institute of Science and Technology, Seoul, Republic of Korea.
