# Supplementary material for: Percentage fractions of urinary di(2-ethylhexyl) phthalate metabolites: Association with obesity and insulin resistance in Korean girls
Source: PLoS One. 2018 Nov 27;13(11):e0208081. doi: 10.1371/journal.pone.0208081 (PMC6258563; doi:10.1371/journal.pone.0208081)
Supplement: S1 Table — (DOCX) [file pone.0208081.s001.docx]

**S1 Table. Relative metabolic rates of di(2-ethylhexyl) phthalate (DEHP) metabolites by obesity and the puberty status.**

|  | **Prepubertal girls (n = 68)** | | |  | **Pubertal girls (n = 69)** | | |  |
| --- | --- | --- | --- | --- | --- | --- | --- | --- |
|  | Normal | Overweight | *P*-value^a^ |  | Normal | Overweight | *P*-value^a^ | *P-*value*^b^* |
|  | n = 35 | n = 33 |  |  | n = 37 | n = 32 |  |  |
| RRM1 | 4.8 ± 1.1 | 4.6 ± 1.1 | 0.790 |  | 5.4 ± 1.1 | 4.8 ± 1.1 | 0.422 | 0.279 |
| **RRM2** | **8.3 ± 1.0** | **7.7 ± 1.0** | **0.016** |  | 7.9 ± 1.0 | 7.8 ± 1.0 | 0.615 | 0.523 |
| RRM3 | 6.1 ± 1.1 | 5.3 ± 1.1 | 0.420 |  | 6.8 ± 1.1 | 6.2 ± 1.1 | 0.458 | 0.313 |

Values are the means ± standard error of the mean.

Abbreviations: RRM, relative rate of metabolism

RRM1 = ([MEHHP] + [MEOHP])/[MEHP]; RRM2 = ([MEOHP]/[MEHHP]) × 10; RRM3 = [MECPP]/[MEHP].

^a^*P*-values for differences between the normal weight and overweight groups.

^b^*P*-values for differences between the prepubertal and pubertal groups.
