# Supplementary material for: Percentage fractions of urinary di(2-ethylhexyl) phthalate metabolites: Association with obesity and insulin resistance in Korean girls
Source: PLoS One. 2018 Nov 27;13(11):e0208081. doi: 10.1371/journal.pone.0208081 (PMC6258563; doi:10.1371/journal.pone.0208081)
Supplement: S2 Table — (DOCX) [file pone.0208081.s002.docx]

**S2 Table**. **Regression analysis of associations between relative metabolic rates of di(2-ethylhexyl) phthalate (DEHP) metabolites and anthropometric indices.**

|  | **Prepubertal girls** | | |  | **Pubertal girls** | | |
| --- | --- | --- | --- | --- | --- | --- | --- |
|  | BMI  percentile | WC (cm) | Body fat (%) |  | BMI  percentile | WC (cm) | Body fat (%) |
|  | β  (95% CI) | β  (95% CI) | β  (95% CI) |  | β  (95% CI) | β  (95% CI) | β  (95% CI) |
| RRM1 | **–**0.69  (**–**3.36 to 1.97) | 0.08  (**–**0.77 to 0.92) | 0.45  (**–**0.45 to 1.36) |  | **–**0.13  (**–**0.63 to 0.37) | **–**0.03  (**–**0.13 to 0.07) | **–**0.01  (**–**0.10 to 0.09) |
| RRM2 | **–7.23**  **(–13.09 to –1.37)*** | **–2.11**  **(–4.00 to –0.21)*** | **–**1.82  (**–**3.89 to 0.24) |  | **–**0.67  (**–**6.64 to 5.29) | **–**1.10  (**–**2.91 to 0.70) | **–**1.17  (**–**2.93 to 0.60) |
| RRM3 | **–**0.63  (**–**2.02 to 0.76) | **–**0.13  (**–**0.56 to 0.32) | 0.07  (**–**0.41 to 0.55) |  | **–**0.07  (**–**0.36 to 0.23) | **–**0.03  (**–**0.12 to 0.06) | **–**0.01  (**–**0.08 to 0.07) |

Regression coefficients were calculated using multivariate linear regression analysis, adjusted for the age, Tanner stage, and height percentile.

RRM1 = ([MEHHP] + [MEOHP])/[MEHP]; RRM2 = ([MEOHP]/[MEHHP]) × 10; RRM3 = [MECPP]/[MEHP].

^*^*P* < 0.05.
