# Supplementary figures and images for: Percentage fractions of urinary di(2-ethylhexyl) phthalate metabolites: Association with obesity and insulin resistance in Korean girls
Source: PLoS One. 2018 Nov 27;13(11):e0208081. doi: 10.1371/journal.pone.0208081 (PMC6258563; doi:10.1371/journal.pone.0208081)

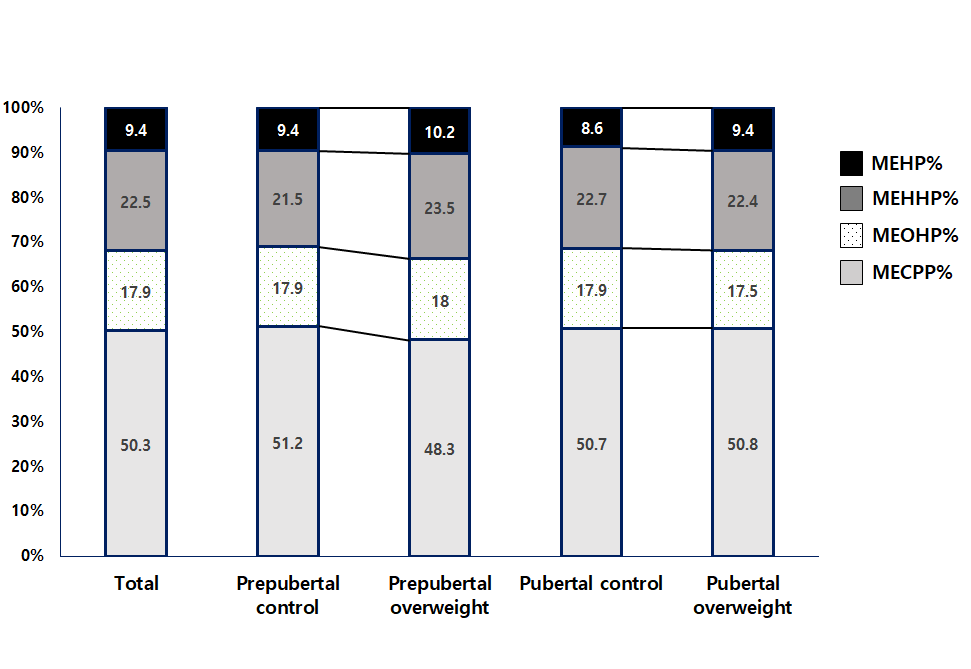

Supplement: S2 Fig — (TIF) [file pone.0208081.s004.tif]
